# Studies of the Sulfonated Hydrogenated Styrene–Isoprene–Styrene Block Copolymer and Its Surface Properties, Cytotoxicity, and Platelet-Contacting Characteristics

**DOI:** 10.3390/polym13020235

**Published:** 2021-01-12

**Authors:** Bin-Hong Tsai, Tse-An Lin, Chi-Hui Cheng, Jui-Che Lin

**Affiliations:** 1 Department of Chemical Engineering, National Cheng Kung University, Tainan 70101, Taiwan; colintsai0324@gmail.com (B.-H.T.); s7161s7161@gmail.com (T.-A.L.); 2 Department of Pediatrics, College of Medicine, Chang Gung University, Chang Gung Memorial Hospital, Taoyuan 33305, Taiwan

**Keywords:** sulfonation, TPE, hydrogenated SEPS, wound dressing, cytotoxicity

## Abstract

Styrenic thermoplastic elastomers (TPEs) consist of styrenic blocks. They are connected with other soft segments by a covalent linkage and are widely used in human life. However, in biomedical applications, TPEs need to be chemically hydrogenated in advance to enhance their properties such as strong UV/ozone resistance and thermal-oxidative stability. In this study, films composed of sulfonated hydrogenated TPEs were evaluated. Hydrogenated *tert*-butyl styrene–styrene–isoprene block copolymers were synthesized and selectively sulfonated to different degrees by reaction with acetyl sulfate. By controlling the ratio of the hydrogenated *tert*-butyl styrene–styrene–isoprene block copolymer and acetyl sulfate, sulfonated films were optimized to demonstrate sufficient mechanical integrity in water as well as good biocompatibility. The thermal plastic sulfonated films were found to be free of cytotoxicity and platelet-compatible and could be potential candidates in biomedical film applications such as wound dressings.

## 1. Introduction

Compared with natural rubbers, thermoplastic elastomers (TPEs) have different attributes: allergen-free, low extractability, ease of sterilization, softness, and clarity for biomedical applications. Among these TPEs, styrene–isoprene–styrene (SIS) triblock copolymers have lower hardness, which is suitable for medical tubing or food package film applications [1]. As SIS contains unsaturated carbon–carbon double bonds in the main chain of the central rubber block, hydrogenation is required to improve its thermal and light stability when used in the biomedical field. After hydrogenation, SIS converted to styrene-(ethylene–propylene)-styrene (SEPS) with improved chemical and physical properties, and these factors have resulted in hydrogenated styrenic block copolymers becoming widely used medical plastics despite the higher cost [2].

Following the use of SEPS in medical tubing, films, or stoppers, biocompatibility has increasingly become a vital factor, especially in tissue-contacting applications. It is generally accepted that material hydrophilicity is among the most important biocompatible attributes; thus, surface modification with hydrophilic functional groups such as hydroxyl or sulfonic is commonly investigated. A common strategy used to improve the hydrophilicity of TPE is to apply high-intensity UV/ozone or oxidants to activate the TPE for further surface-grafting processes [3,4,5,6]. Studies have addressed the TPE surface patterning of hydrophilic polymer brushes and architectural construction are also efficient ways to fabricate biocompatible and bioactive surfaces [7,8]. In previous studies, grafting with sulfonic groups on biomaterial has been considered an effective method to improve biocompatibility [9,10,11,12,13,14], and most research has only focused on surface-level modifications [15,16]. However, regarding soft SEPS, few investigations have addressed the issue of losing structural integrity during swelling in a water solution after harsh oxidative chemical treatments [17]. Therefore, the balance between surface hydrophobicity and structural integrity has become challenging in the development of SEPS biomedical applications. Recent developments of a new hydrogenated styrenic block copolymer (HSBC) compound regarding water treatment could have led to a new SEPS approach in the biomedical field. To improve the hydrophilicity of HSBCs without losing their physical integrity, Kraton LCC developed a controllable sulfonated pentablock styrenic copolymer (s-PBC), commercialized as Nexar^TM^. Functionalization with sulfonic groups in Nexar^TM^ allows this polymer to achieve an excellent balance between various applications, mainly due to its block molecular structure and functionalization with sulfonic hydrophilicity and mechanical stability [18]. The unreacted *tert*-butyl functional end group prohibits the resulting block copolymers from dissolving in water, and sufficient mechanical strength of swollen copolymer films is also maintained [19]. Until now, this methodology has only been applied to the industrial water treatment field; to the best of our knowledge, no one has investigated the biocompatibility of sulfonated HSBCs.

In our previous work, we found that the sulfonation of blends of polypropylene/*tert*-butyl SEPS demonstrated physical stability as well as biocompatible performance in terms of mechanical intactness, low platelet adhesion, and being free of cytotoxicity [20]. We also observed that the mechanical property of blends of sulfonated PP/*tert*-butyl SEPS with different mixing ratios was brittle, and the brittleness of blends of sulfonated PP/*tert*-butyl SEPS film can be reduced dramatically with the aid of mineral oil as an additional plasticizer. The aim of the present work is to systematically study the effect of the sulfonation degree of a neat *tert*-butyl SEPS copolymer to form biocompatible elastomers without any plasticizer additive. A triblock copolymer, SIS, was successfully polymerized via anionic polymerization. The hard segment of SIS was composed of specific ratios of styrene and *tert*-butyl styrene to control the degree of sulfonation. SIS is an unsaturated block copolymer with carbon–carbon double bonds in the molecular chain, leading to low stability under high sterilization temperature or UV radiation. To overcome this issue, the SIS triblock copolymer was treated with the hydrogenation process, saturating the existing double bonds and giving the resulting styrene-ethylene–propylene-styrene (SEPS) triblock copolymer better thermal properties such as temperature resistance.

After the hydrogenation process, a selective sulfonation process was conducted, introducing the hydrophilic sulfonic functional groups into the SEPS triblock copolymer. The degree of sulfonation of SEPS was controlled as a function of the ratio of the *tert*-butyl functional group and acetyl sulfate. The sulfonated SEPS films demonstrated a promising balance of hydrophilicity and structural integrity, which remained physically intact in an aqueous solution. Finally, the cytotoxicity and platelet-contacting results suggest that sulfonated SEPS (i.e., sulfonated hydrogenated SIS) exhibits great potential as future candidates for wound-dressing or tissue-engineering applications.

## 2. Materials and Methods 

### 2.1. Materials

All reagents used were of analytical grade and carefully demoistured. The essential materials used in this experiment, which were styrene, 4-*tert*-butyl styrene, and isoprene, were obtained from Alfa Aesar (Loughborough, UK). The N-butyllithium solution, cobalt 2-ethylhexanoate solution, and acetic anhydride were supplied by Sigma-Aldrich (St. Louis, MO, USA). Cyclohexane (J. T. Baker, Allentown, PA, USA), sulfuric acid (J. T. Baker, USA), 1,2-dichloroethane (Alfa Aesar), and triethylaluminum were used as received. Bovine serum albumin (BSA), sodium dodecyl sulfate (SDS), and phosphate-buffered solution (PBS, 0.1 mol/L, pH = 7.4) were provided by Thermo Fisher (Waltham, MA, USA). 

### 2.2. Analytical Methods

^1^H NMR analysis of all the samples was recorded on a Bruker AV-500 (Berlin, German) with CDCl3 as the solvent and tetramethylsilane as an external reference. All the spectra were referenced at δ = 7.25 ppm for 1 H to CDCl_3_. FTIR spectra were recorded with a Varian 630-IR instrument (Palo Alto, CA, USA) with a resolution of 4 cm^−1^ in the transmission mode, in which the samples were cast onto a KBr disk from a solution of the elastomer dissolved in chloroform. Gel permeation chromatography (GPC) was obtained using a Waters 150-C ALC/GPC instrument (Millipore, Burlington, MA, USA) equipped with a set of ViscoGEL I-Series Columns. The surface morphology of sample films was investigated using a JEOL JSM-6700F HR-FESEM (Tokyo, Japan). The polymeric samples in the form of thin films, before analysis, were coated with gold in a sputter coater to achieve a conducting surface and were analyzed at an accelerated voltage (potential) of 10 kV. 

### 2.3. Synthesis of the SIS Triblock Copolymer

**Scheme 1:**
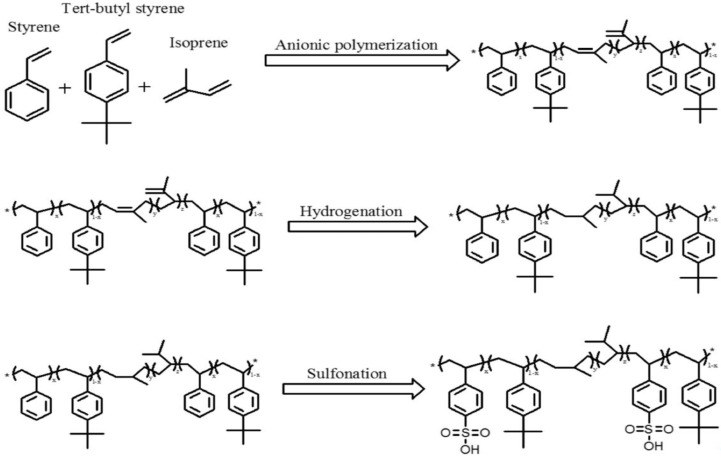
The synthesis process of styrene-(ethylene–propylene)-styrene (SEPS) and sulfonated SEPS.

A series of SIS triblock copolymers, named S5T5, S6T4, S7T3, S8T2, and S9T1, were prepared in brief according to the process described in Scheme 1. The feed ratio of the two monomers styrene and 4-*tert*-butyl styrene was altered for different copolymers, and the amount of isoprene was fixed (Table 1).

In a 250 mL round-bottomed flask, a specific amount of styrene and 4-*tert*-butyl styrene was dissolved in 80 mL of cyclohexane at room temperature. Great attention must be taken when conducting moisture-sensitive anionic polymerization. A 0.3 g amount of *tert*-BuLi dissolved in 10 mL of cyclohexane was added dropwise with a syringe over a time of 1 min. The reaction mixture was maintained at 53 °C, and the reaction was continued for 12 h under constant stirring until the solution turned dark red. A 10 g amount of isoprene then was added to the reaction mixture and kept in the reaction for 5 h, after which the same specific amount of styrene and 4-*tert*-butyl styrene was added for an additional 12 h reaction and precipitated in 1 L of a methanol/acetone co-solvent for the termination of the reaction. The precipitated polymer was washed several times with methanol. The polymer was then dried in a desiccator under vacuum at room temperature.

### 2.4. Hydrogenation of the SIS Block Copolymer

SIS copolymers were hydrogenated according to previous procedures [21,22,23,24] with some modifications. The hydrogenation process was conducted under an argon atmosphere, and all Schlenk flasks were filled with argon after high-temperature heating. A triethyl aluminum/cobalt 2-ethyl hexanoate complex was used as a homogeneous hydrogenation catalyst. Firstly, a Schlenk flask was charged with 20 mL of the cobalt 2-ethylhexanoate cyclohexane solution (1.34 mmol) followed by cooling in a liquid nitrogen bath. Secondly, the cobalt 2-ethylhexanoate cyclohexane solution was degassed by a freeze–pump–thaw cycle two times and injected with 4.8 mL of triethyl aluminum via syringe for the reaction for 1 h until the solution turned from purple-blue to dark blue.

Thirdly, an argon-purged reactor was loaded with cyclohexane (140 mL) and SIS (6 g) and stirred until the SIS was fully dissolved. Later, 15 mL of a catalytic complex was added into the reactor, which was then closed and purged with argon. The reactor was heated to 60 °C and purged with hydrogen, and the hydrogen pressure was increased up to 6 bar. Six hours later, the catalyst was deactivated by the addition of a citric acid solution (50 mmol/L). The copolymer was precipitated in acetone/ethanol, washed and redissolved in cyclohexane, reprecipitated, and dried under vacuum conditions for the final prepared SEPS block copolymer.

### 2.5. Sulfonation of the SEPS Block Copolymer

Acetic anhydride (7.63 mL) and 1,2-dichloroethane (39.57 mL) were placed in a two-necked-bottomed flask at 10 °C, followed by the addition of sulfuric acid (15 mL) to obtain acetyl sulfate. Hydrogenated SIS, the SEPS (2.5 g), was dissolved in 1,2-dichloroethane and acetyl sulfate (3 mL) was added. The sulfonation reaction was carried out in an oil bath at 50 °C for 2.5 h. The sulfonated copolymer was dropped into boiling water, giving the crude product. Finally, the degree of sulfonation was determined by titration. In brief, 0.1 g of dehydrated sulfonated polymer was dissolved in about 10 mL of a toluene/methanol mixture (9:1 vol). A standard 0.1 N solution of sodium potassium was first diluted 10 times with methanol and used to titrate the polymer solution, with phenolphthalein as an indicator.

### 2.6. Streaming Potential

The streaming potential of the flat substrate was determined by the SurPass Electro-Kinetic analyzer (Anton Paar KG, Graz, Austria). To mimic the physiological conditions, phosphate-buffered saline (PBS) at pH 7.4 was used as the electrolyte solution. When the electrolyte solution flowed over the flat substrates under moderate pressure, the streaming potential was measured by two Ag/AgCl electrodes, placed at the inlet and outlet of the fluid cell. 

### 2.7. Cytotoxicity Assay

The cytotoxicity evaluation of the SEPS (hydrogenated SIS) films was processed according to the ISO10993-5 and ISO-10993-12 standard testing methods. NIH-3T3 fibroblast cells were used for the biocompatibility assay on these SEPS substrates. Briefly, the NIH 3T3 fibroblast cells were seeded in a 24-well plate at a density of 6 × 10^4^ cells/well and incubated in Dulbecco’s Modified Eagle’s medium (DMEM). The SEPS films were cut to 8 mm thick disks (~1.3 g each) and immersed in a 24-well culture plate with DMEM for 24 h. After 24 h, the culture medium for the NIH 3T3 fibroblast cells was replaced by the medium obtained from the SEPS incubation/extraction. The cells were further incubated for another 24 h, and the number of viable cells was determined using an automated cell counter (TC20, Bio-Rad, Hercules, CA, USA). Briefly, the cells were trypsinized to form cell suspensions and mixed with trypan blue (0.4%, Gibco, USA) at a volume ratio of 1:1. The well-mixed solution was added to the cell counting slide, and the viable cell number was calculated. For the direct-contact cytotoxicity assay, the NIH 3T3 fibroblast cells were seeded in a 24-well plate, in which SEPS films with a film thickness of about 1 mm (~0.16 g each) were preinserted into each well at a density of 6 × 10^4^ cells/well and incubated in DMEM for 24 h.

### 2.8. In Vitro Platelet Adhesion Test

The sulfonated and nonsulfonated films were placed in a tissue culture plate and incubated with Hepes–Tyrode’s solution for 1 h before use. The fresh platelet-rich plasma (PRP) was obtained from the local Tainan Blood Donation Center. The Hepes–Tyrode’s solution was removed, and 5 mL of the PRP solution was introduced on each film and incubated for 1 h at 37 °C. The adhered platelets on the films were washed with Hepes–Tyrode’s solution three times and fixed by the Hepes solution containing 2% (v/v) of glutaraldehyde for 30 min. Finally, the films were dehydrated in ascending ethanol/water mixtures (25 vol %, 50 vol %, 75 vol %, 100 vol % ethanol) for 3 min during each step. Finally, the substrates were dried with CO_2_ critical point drying and were immediately sputter-coated with Au for further SEM morphological analyses. The number of adhered platelets on the films was calculated from several SEM pictures of the same film at a magnification of 3000×. The number of activated (i.e., not in a round shape) adhered platelets was also examined [25,26]. 

## 3. Results

### 3.1. Material Characterization

In this study, the acronym “S_x_T_y_” denotes the nomenclature of copolymers prepared from styrene (S) and 4-*tert*-butyl styrene (T); x and y stand for the weight ratio of styrene and 4-*tert*-butyl styrene, respectively (Table 1). In order to obtain a well-defined polymeric structure, anionic polymerization was selected for the synthesis of a specific ratio of styrene/4-*tert*-butyl styrene with isoprene [27]. 

**Figure 1:**
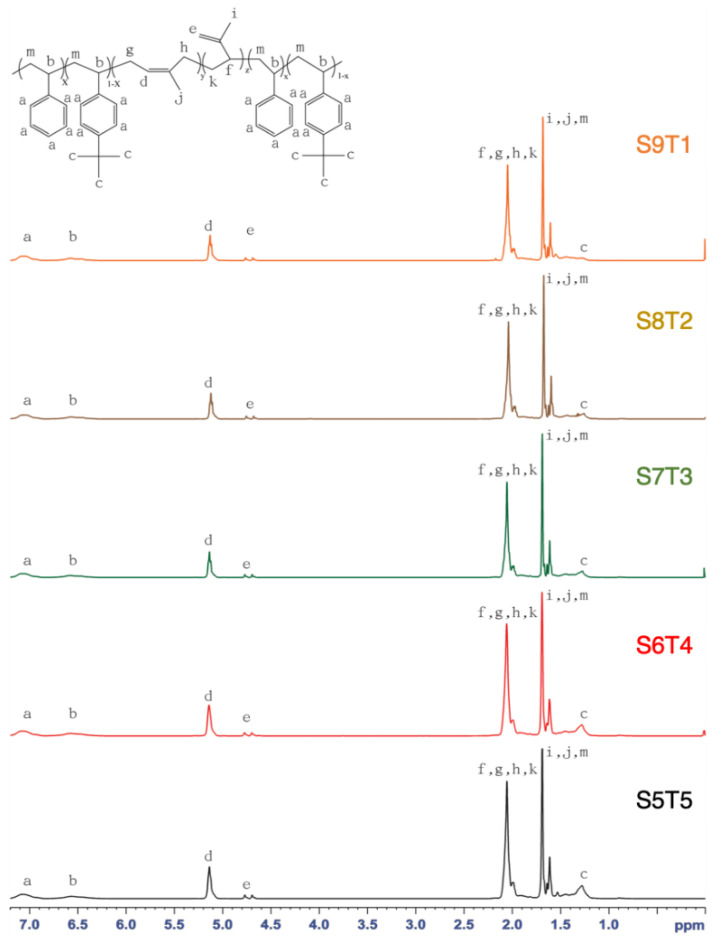
^1^H-NMR spectra of styrene–isoprene–styrene (SIS) triblock copolymers of different feed ratios.

The NMR spectra of each block of copolymers with different feed weight ratios of styrene and 4-*tert*-butyl styrene are shown in Figure 1. The ^1^H NMR spectra of SIS showed characteristic signals of different protons at δ values of 7.05–6.57 ppm (aromatic protons on phenyl rings), 5.1 ppm (vinyl protons of the 1,4-isoprene unit), and 4.7 ppm (vinyl protons of the 3,4-isoprene unit). A 1.85–2.10 ppm range addressed the methylene proton of the 1,4-isoprene and 3,4-isoprene units, and the successful protection of the 4-*tert*-butyl group was confirmed by the existence of the characteristic signal of the 4-*tert*-butyl group at 1.3 ppm [28]. The atomic ratio of each block was calculated by the integrated area of the characteristic peak [29,30]. Based on Figure 1, the peak integration area of 1.3 ppm decreased with the increment of the styrene ratio from 5:5 to 9:1. Specific proton chemical shifts showed that the reaction was well controlled. The molar ratio of each block was calculated using Equation (1):

(1) $$\begin{array}{c} \mathrm{styrene}:\;tert-\mathrm{butyl}\;\mathrm{styrene}:\;1,4\;\mathrm{isoprene}:\;3,4\;\mathrm{isoprene}\\ =\frac{A-4\times \frac{C}{9}}{5}:\frac{C}{9}:E:\frac{D}{2} \end{array}$$

where *A* represents the peak area of nine protons on the styrenic aromatic ring (integral area under Peak A), including five protons of styrene and four protons of *tert*-butyl styrene; *C* expresses the nine protons of the *tert*-butyl group (integral area under Peak C); *D* addresses the single unsaturated proton of 1,4-isoprene (integral area under Peak D); *E* indicates two unsaturated protons of 3,4-isoprene (integral area under Peak E). The structural characteristics of the different SIS copolymers are summarized in Table 1. It is noted that the experimental styrene/*tert*-butyl styrene weight ratio increased with the amount of styrene added. However, all styrene/*tert*-butyl styrene weight ratios were lower than their theoretical counterparts, likely resulting from higher *tert*-butyl styrene reactivity than styrene. All experimental styrenic block percentages (mole %) were similar to each other and were higher than the theoretical counterparts.

**Figure 2:**
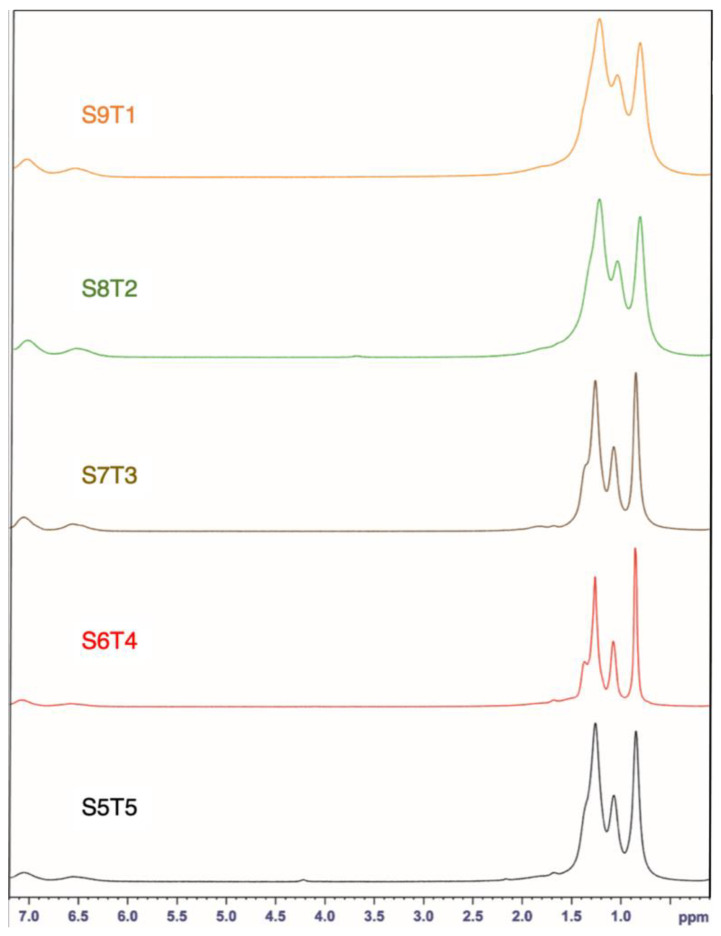
^1^H-NMR spectra of different SIS copolymers after hydrogenation.

After hydrogenation, the chemical shifts at 4.7 and 5.1 ppm, assigned to the vinyl protons of the 3,4-isoprene and 1,4-isoprene units, respectively, disappeared (Figure 2). Thus, we could confirm that the double bonds in the soft segments were fully saturated.

Since the sulfonated block copolymers were insoluble in standard deuterium solvents, the NMR analyses for these specimens were not executed. The degree of sulfonation (DS, the ratio of the number of styrene units attached with the sulfonated group compared to the total number of styrene unit), was calculated using Equation (2)

(2) $$\mathrm{DS}\;\left(\mathrm{mole}\;\%\right)=\frac{V\times C}{V\times C+\frac{m-V\times C\times M_{1}}{M_{2}}}\times 100\%$$

where *V* represents the volume of methanolic KOH titrant consumed, and *C* denotes the molar concentration of KOH solution. *m* addresses the weight of styrene in the block copolymer, where *M*_1_ and *M*_2_ represent the molecular weight of *tert*-butyl styrene and styrene.

The DS for sulfonated samples sS5T5, sS6T4, sS7T3, sS8T2, and sS9T1 were 2.82 ± 0.29%, 1.68 ± 0.11%, 2.59 ± 0.28%, 0.78 ± 0.14%, and 1.10 ± 0.32%, respectively. Statistical analyses revealed that the order of DS was as follows: S5T5–sS7T3 > sS6T4 > sS9T1–sS8T2. The reason behind the difference in the degree of sulfonation might be linked to the different ratios of styrene and 4-*tert*-butyl styrene. In the S5T5 sample, the styrene units in the hard segment could be well dispersed, and the sulfonic functional groups could be introduced without high hindrance. Similarly, the higher amount of 4-*tert*-butyl styrene that could be sulfonated (Scheme 1) would also lead to a higher DS noted. Hence, the difference in the hard segment morphology and the amount of 4-*tert*-butyl styrene available for sulfonation could synergistically affect the DS noted.

**Figure 3:**
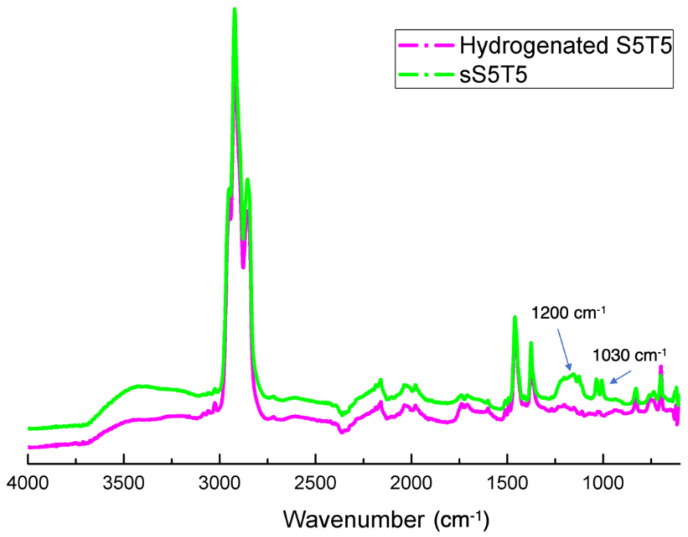
ATR-FTIR spectra of the hydrogenated S5T5 and sulfonated hydrogenated S5T5 (sS5T5).

The sulfonation of the polystyrene block was further confirmed by ATR-FTIR analysis (Figure 3). A doublet positioned at 1200 cm^−1^ and a band at 1030 cm^−1^, corresponding to the antisymmetric and symmetric stretching vibrations of SO_3_^−^, respectively, [31] were noted, implicating the success of sulfonating the hydrogenated specimens.

**Figure 4:**
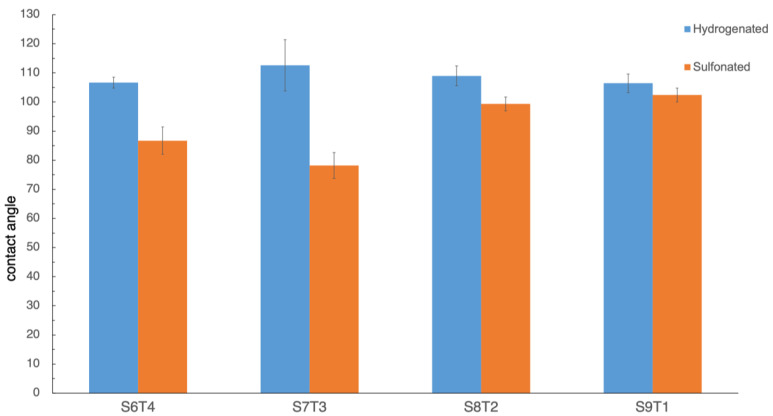
Contact angle results of the hydrogenated and sulfonated hydrogenated SIS samples.

The contact angle of hydrogenated and sulfonated hydrogenated SIS membranes with different degrees of sulfonation is illustrated in Figure 4. As shown in Figure 4, the hydrogenated SIS membrane showed a high water contact angle of about 106°, which is close to the values reported by Patiño et al. [28]. The water contact angle of the sulfonated membranes decreased progressively by increasing the degree of sulfonation, owing to the incorporation of hydrophilic sulfonic groups. Interestingly, for the sS5T5 membrane with the highest degree of sulfonation, the membrane was falling apart after contact with water, and, hence, it was not used for subsequent analyses needed for water/PBS contact for a while. 

Water uptake was determined by immersing the sample membranes into the deionized water at room temperature for 12 h, removing any excess water on the surface, and finally weighing them on a microbalance. The water uptake was:

$$W\left(\%\right)=\frac{Ww-Wd}{Wd}\times 100$$

where *Wd* and *Ww* are the mass of the dry sample and the mass of the water-swollen sample, respectively. It was noted that the water uptake values for the sulfonated samples of sS6T4, sS7T3, sS8T2, and sS9T1 were 30.19 ± 2.27%, 40.63 ± 1.79%, 18.91 ± 0.21%, and 8.47 ± 0.65%, respectively. These values correlated with the degree of sulfonation of the tested specimens.

**Figure 5:**
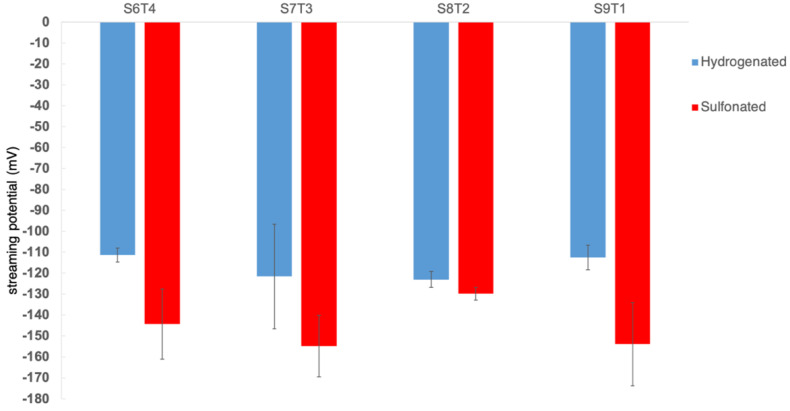
Streaming potential of the hydrogenated and sulfonated hydrogenated SIS samples.

The surface charge density could have played an important role when the material was in contact with the physiological environment. The streaming potential of the nonsulfonated hydrogenated SIS membranes and sulfonated membranes is shown in Figure 5. With the incorporation of anionic sulfonic functional groups, the membrane surface became more negatively charged compared to the nonsulfonated groups, except S8T2. However, there were no significant differences among the sulfonated samples, except sS8T2, on which the streaming potential was more statistically positive.

### 3.2. Cytotoxicity Test

**Figure 6:**
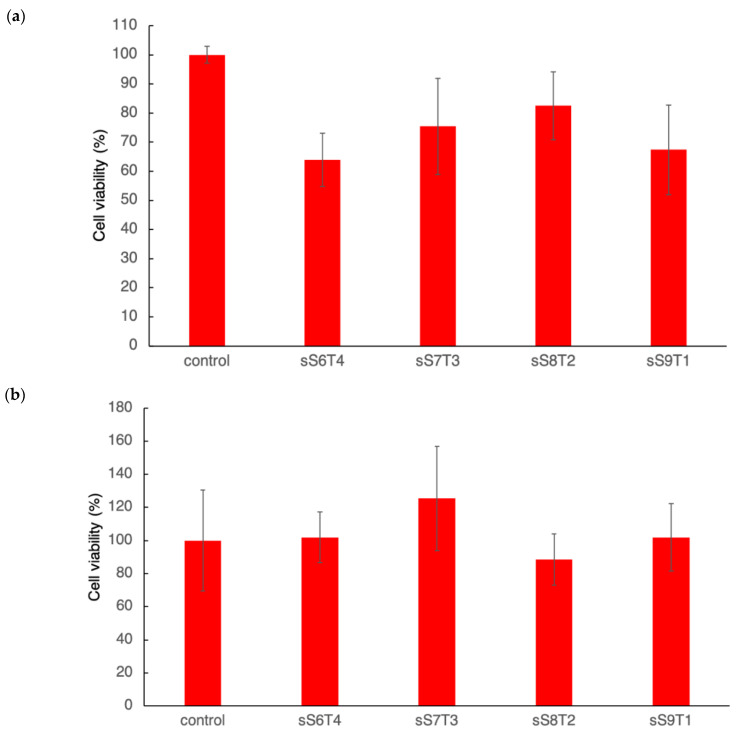
Cytotoxicity assay on different sulfonated copolymers by (**a**) the extracts and (**b**) direct contact.

Cell viability testing was required for the material intended for biomedical applications. Following the guidance of ISO 10993-5 and ISO 10993-12, which focused on the cell viability testing on the extracts of the sulfonated copolymers (Figure 6A) and direct contact with the copolymers (Figure 6B). The difference in cell viability values between these two assays could be attributed to the difference in the film thickness used: 8 mm for the extract assay and 1 mm for the direct-contact experiment. Nevertheless, the film thickness of the sulfonated copolymers will be within this tested range for future applications. The results have shown all sulfonated materials presented over 70% cell viability value, either by testing the extract or by direct contact, required by the ISO standard for nontoxic materials. 

### 3.3. In Vitro Platelet Adhesion Testing

**Figure 7:**
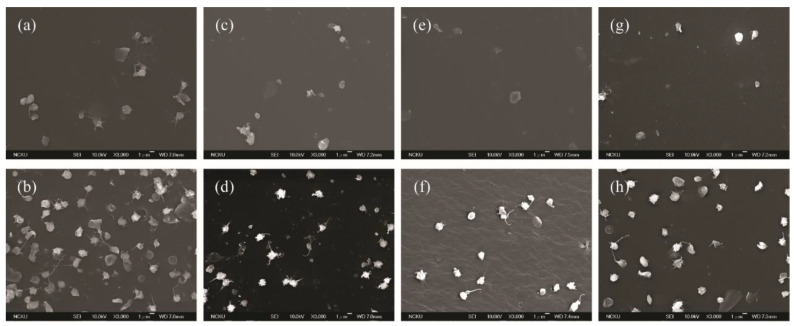
SEM micrographs (3000×) for the platelets adhered onto the hydrogenated (**a**) S6T4, (**c**) S7T3, (**e**) S8T2, (**g**) S9T1 and sulfonated hydrogenated (**b**) sS6T4, (**d**) sS7T3, (**f**) sS8T2, and (**h**) sS9T1 (scale bar: 1 µm).

**Figure 8:**
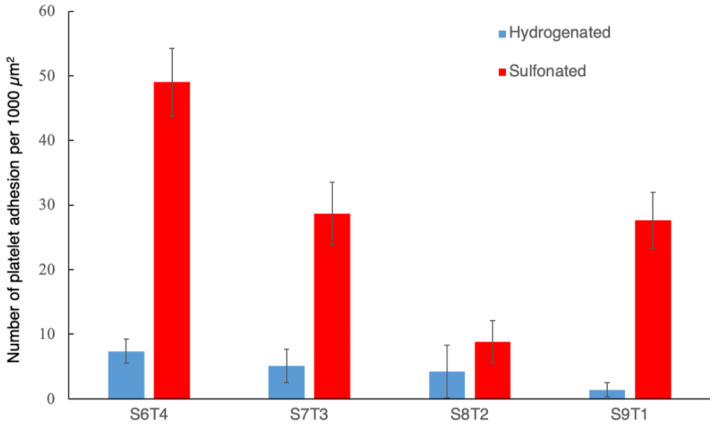
Platelet adhesion density on the hydrogenated and sulfonated block copolymers.

**Figure 9:**
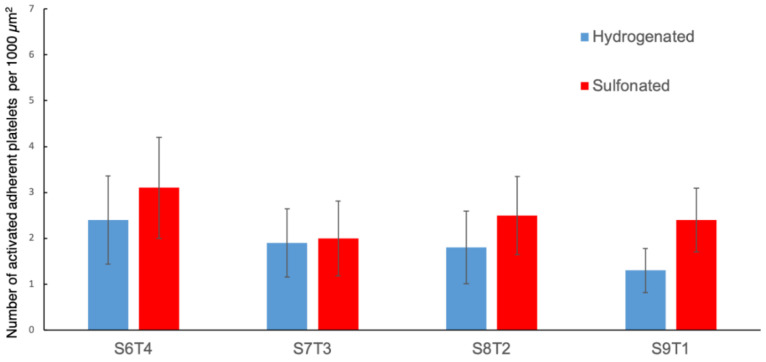
Number of activated (i.e., not in a round shape) adherent platelets per 1000 µm^2^ on the hydrogenated and sulfonated block copolymers.

The adhesion and activation of platelets on material surfaces could initiate thromboembolic complications. Therefore, there is a need to conduct in vitro platelet adhesion tests to evaluate the biocompatibility of our samples. Figure 7 and Figure 8 show the results of the in vitro platelet adhesion test on the hydrogenated and sulfonated hydrogenated copolymer surfaces. All sulfonated samples demonstrated a higher amount of platelet adhesion density than the unsulfonated counterparts. Nevertheless, most of the adhered platelets remained unactivated or only slightly activated (Figure 9).

Such an increase in platelet adhesion density but with an unactivated morphology was also noted on the sulfonate-incorporated polyether polyurethane [32] as well as our earlier study on the N,O-sulfated chitosan [33]. This is likely due to the surface-negative charge at the physiological incubation condition, which changed the adsorbed protein composition and conformation, resulting in increased platelet adhesion while not being activated. It was of note that the sulfonated S8T2 presented the lowest platelet adhesion density and had the least negative streaming potential (Figure 5). This further substantiated the importance of surface charge when coming into contact with blood/platelets.

## 4. Conclusions

By controlling the weight ratio of *tert*-butyl styrene in the hydrogenated SIS block copolymers, sulfonated copolymeric films were demonstrated to have hydrophilic swollen characteristics without losing their physical intactness. Biological assays showed these sulfonated samples are noncytotoxic. Although the platelet adhesion assay indicated these sulfonated films could not effectively decrease the platelet adhesion density, the adhered platelets were still not activated and, hence, the least thrombogenic-prone when in contact with the whole blood. Further studies will be required for optimizing the physical performance of sulfonated hydrogenated SIS as a potential alternative for wound-dressing applications.

## Figures and Tables

**Table 1:** Composition of different SIS copolymers.

	S5T5	S6T4	S7T3	S8T2	S9T1
Styrene feeding weight (g)	1.0	1.2	1.4	1.6	1.8
*tert*-butyl styrene (tbs) feeding weight (g)	1.0	0.8	0.6	0.4	0.2
Isoprene feeding weight (g)	5.0	5.0	5.0	5.0	5.0
Styrene/tbs (experimental weight ratio) ^a^	0.67	0.79	0.91	1.05	1.16
Styrenic block (theoretical mole %)	0.18	0.18	0.19	0.20	0.20
Styrenic block (experimental mole %) ^a^	0.39	0.36	0.37	0.37	0.34
1,4 isoprene/3,4 isoprene (molar ratio) ^a^	15.45	14.02	13.37	14.24	15.05

^a^ Calculated from ^1^H NMR.

## Data Availability

The data presented in this study are available on request from the corresponding author.
